# Case Report of Multiple Tracheostomy Revisions due to Persistent, Recurrent Cuff Leak

**DOI:** 10.1155/2015/379397

**Published:** 2015-07-09

**Authors:** Jian P. Azimi-Bolourian, Issa A. Hanna, George W. Williams

**Affiliations:** ^1^Department of Anesthesiology, The University of Texas Medical School at Houston, Houston, TX 77030, USA; ^2^Department of Oral Maxillofacial Surgery, The University of Texas School of Dentistry at Houston, Houston, TX 77030, USA; ^3^Departments of Anesthesiology and Neurosurgery, The University of Texas Medical School at Houston, Houston, TX 77030, USA

## Abstract

This case is a patient with amyotrophic lateral sclerosis who was unable to be separated from mechanical ventilator support and required a tracheostomy. The patient underwent an initial open tracheostomy utilizing flexible fiberoptic tracheoscopy (FFT) in the operating room (OR). Subsequently, he developed recurrent leaks in the tracheal tube cuff requiring multiple trips back to the operating room. The recurrent cuff leak occurred following each tube placement until the etiology of the leak was discovered during the fourth procedure. In the fourth procedure, the wound was explored more extensively, and it was found that there was a sharp, calcified, aberrant fragment of a tracheal cartilage ring protruding into the tracheal lumen, which was damaging the cuff of each tube. This fragment was not visible by multiple FFTs, nor was it visible in the wound by the surgeons until wider exploration of the wound occurred. The cartilage fragment was ultimately excised and the patient had no further cuff leaks. Aberrant tracheal cartilage should be on the differential diagnosis for cuff leaks subsequent to surgical tracheostomy (ST) or percutaneous dilatational tracheostomy (PDT).

## 1. Introduction

This case report is that of a 61-year-old male with a past medical history of amyotrophic lateral sclerosis, who presented with hypoxic respiratory failure requiring intubation and mechanical ventilation. He underwent a ST in the OR for failure to wean from the ventilator. Due to recurrent cuff leaks, he underwent multiple revisions in the OR.

## 2. Case Description

Our patient is a 61-year-old 90 kg 177.8 cm male with a history of coronary artery disease and amyotrophic lateral sclerosis who was admitted to the intensive care unit for ventilator support. On arrival to the emergency department he was profoundly hypoxic and was emergently intubated. He was subsequently transferred to the intensive care unit. On initial evaluation the patient was alert and was able to follow commands and communicated via writing. His overall strength was poor, 2/5 in both his hands and feet with minimal ability to lift his proximal arms and legs. His cranial nerves were all grossly intact. His level of function prior to admission was heavily dependent on assistance for activities of daily living. He was, however, able to talk and swallow without difficulty and was receiving no respiratory support.

**Figure 1:**
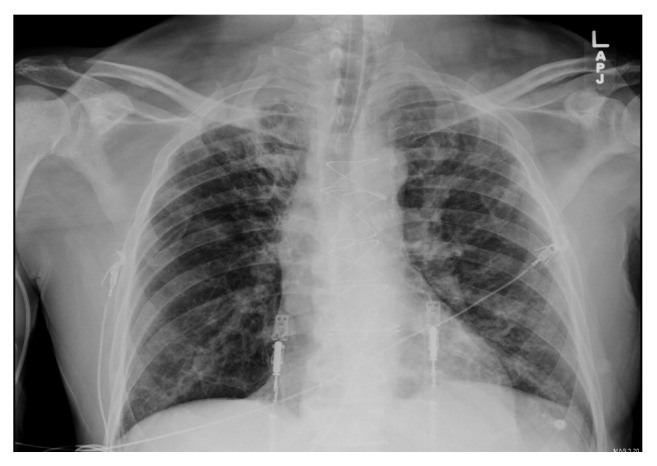
Preoperative chest X-Ray (CXR).

Subsequent to his admission to the ICU he met parameters for extubation during a trial of spontaneous breathing, and the endotracheal tube was removed. He failed spontaneous ventilation and was reintubated within 2 hours following extubation. Over the next three days, multiple attempts were made to wean the patient from the ventilator; however, he failed multiple trials of spontaneous breathing and could not be extubated. The decision was made among the ICU team, the patient, and the family to proceed with tracheostomy insertion. The Oral Maxillofacial Surgery (OMFS) service was then consulted. See Figure 1 for the preoperative chest X-Ray (CXR).

This patient was taken to the operating room by the OMFS service. An open tracheostomy was performed with placement of a 6.0 cuffed Shiley tracheostomy tube, under flexible fiberoptic tracheoscopy (FFT) assistance, without difficulty. It was noted in the operative report that the patient had hardening and calcification of the first, second, and third tracheal cartilages. It was also noted that he had a short neck and a posterior trachea. No tracheal abnormalities were observed during FFT after tracheostomy insertion. Within a few hours of the patient returning to the ICU a cuff leak developed. The OMFS service was consulted and he was emergently taken back to the operating room a second time with a concern that the tracheal tube might have become displaced. Intratracheal placement of the leaking tube was confirmed by FFT. Again no tracheal abnormalities were observed. A Cook Exchange Catheter (CEC) was placed through the tracheostomy tube and the damaged tube was withdrawn. A 6.0 cuffed endotracheal tube was placed over the CEC. Verification of tracheal placement was confirmed with both end tidal CO2 and FFT.

Placement of a longer 6.0 cuffed Shiley was attempted but aborted due to difficulty in placement. A reinforced 6.5 endotracheal tube was then placed over the CEC. Tracheal placement of the reinforced endotracheal tube was confirmed by ETCO2 and FFT with no noted tracheal abnormalities. The patient was returned to the ICU.

**Figure 2:**
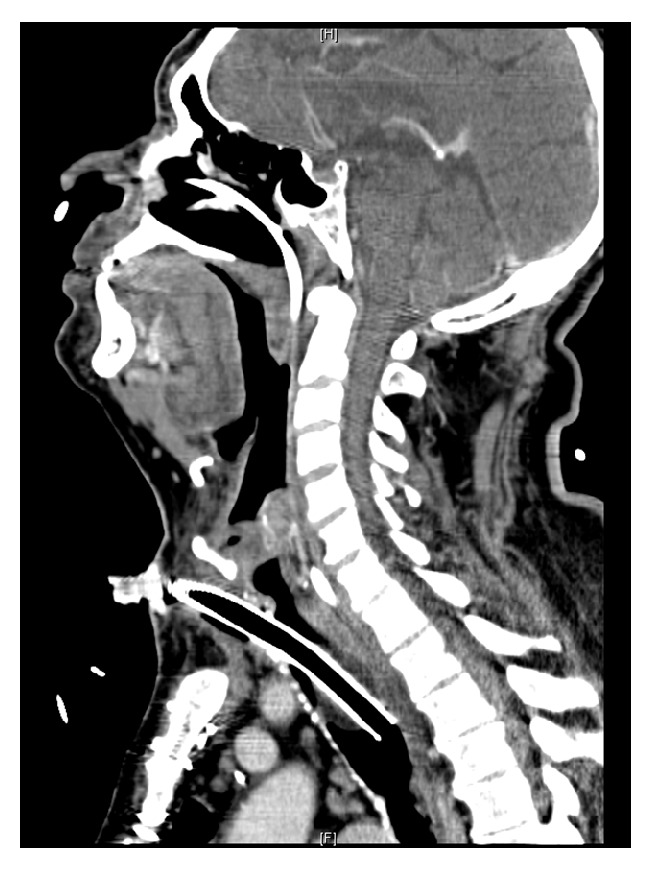
Postoperative Neck Soft Tissue CT revealing diffuse tracheal calcification.

Two days later the patient was noted to have gurgling at the tracheostomy site and was taken to the operating room a third time. The 6.5 reinforced ETT was removed over a bougie and a 6.0 cuffed Shiley with proximal extension was placed and sutured to the skin. Good ventilation was confirmed. During FFT, no tracheal abnormalities were noted. Later that same day gurgling was again noted and our patient returned to the operating room a fourth time to replace the leaking tube. The tube was exchanged over a Pediatric Cook Exchange Catheter. Examination of the cuff of the removed tube revealed a leak from the left anterior aspect of the cuff. During this procedure the OMFS surgeon explored the wound further to attempt to delineate a cause for the multiple cuff leaks. In order to gain access to trachea, Army-Navy retractors were down in the surgical site. With further exposure of the surgical site with a Debakey forcep, a 10 mm by 4 mm piece of sharp calcified cartilage was discovered protruding into the tracheal lumen in a location consistent with the previously noted cuff damage. The team was not able to obtain an image of the protruding cartilage. Additionally, the ruptured cuff was tested for bubbles in an emesis basis intraoperatively, though images were not obtained. The cartilage was excised, and an 8.0 Bivona adjustable tracheostomy tube was placed without further difficulty. The patient returned to the ICU. On POD #5 a Neck Soft Tissue CT, which was obtained in order to rule out complications from the repeated tracheostomy attempts, demonstrated increased diffuse tracheal calcification (see Figure 2). He experienced no further complications with the tracheostomy tube during his hospitalization and was subsequently discharged for further rehabilitation.

## 3. Discussion

Our patient displayed a previously unreported complication of open tracheostomy and passage of a cuffed tube into the tracheotomy, requiring four subsequent procedures. A small but initially invisible (by FFT or direct vision) aberrant sharp calcified outgrowth of tracheal cartilage protruding into the tracheal lumen was responsible for damage to the cuff of the first 3 tracheostomy tubes placed. This cartilage was excised and the balloon on the final tracheostomy tube remained intact after final placement. In this case, the OMFS team elected to place a 6.0 tracheostomy tube which is uncustomary to the standard 8.0 tracheostomy tube normally placed for adult males meeting this patient's physical parameters and ventilation requirements from a pulmonary toilet and weaning perspective. A smaller tube was placed because of the concern of placement difficulty based on neck anatomy with consideration of the tracheal calcification noted by the surgeon at the time of initial placement. In retrospect, this patient had significant tracheal calcification on soft tissue CT of the neck, but this imaging was not completed until after the repeated tracheostomies. CXR was unrevealing of any significant calcification and even though there was continuous visualization with FFT no cartilage was noted during initial placement. The only means of detecting the cartilage was via direct visualization on reexploration. During initial placement of a tracheostomy tube, tracheal calcification can be palpated or visualized, as it was in this case. Tracheal calcification is plausibly necessary to make a fragment sharp and resilient enough to achieve cuff puncture and therefore can be considered a risk factor for tracheostomy tube cuff rupture.

Tracheostomies are commonly performed in critically ill patients and may be placed surgically or percutaneously. The complication rate is generally lower than 3% [1]. First described in 1909, ST may result in several life-threatening complications, including tension pneumothorax, subcutaneous emphysema, and arterial-tracheal fistula [2–4]. Tracheal ring fracture has been noted to occur during open tracheostomy, though this occurrence is rare [5]. Tracheal stenosis as early as 15 days has been reported following ST [6]. Infection is also a potential complication of ST and PDL. Antonelli et al. demonstrated that >70% of patients presenting for tracheostomy had positive aspirate cultures [7]. Repeat blood cultures drawn three hours postoperatively revealed bacteremia in 18% of translaryngeal tracheostomies and 19% of surgical tracheostomies. Nearly half of the positive blood cultures were identical species to the tracheal aspirates, and almost one-third of patients with bacteremia eventually had severe sepsis. Long-term complications include phonetic or respiratory problems.

Additionally, PDT is commonly used in the ICU environment [8]. Advantages to percutaneous techniques include speed of procedure, less personnel, smaller skin incision, less tissue trauma, lower wound infection, and peristomal bleeding. However, percutaneous tracheostomy has a statistically higher likelihood of decannulation and obstruction when compared with open tracheostomy. Additionally, stomatitis is another complication of tracheostomy that can be reduced with the percutaneous approach.

More rare complications of PDL include aortic arch laceration and tracheal wall tear [9, 10]. In both PDT and ST, bronchoscopy may be useful in diagnosing and managing some of the observed injuries, thereby facilitating treatment.

In conclusion, critical care medicine physicians should be aware of the complications associated with tracheostomy and should consider tracheal cartilage abnormalities when a cuff leak occurs soon after tracheostomy insertion. Although FFT did not reveal the tracheal abnormality in our patient, its use should be considered during ST as it may decrease the complication rate.
